# Early cellular and synaptic changes in dopaminoceptive forebrain regions of juvenile mice following gestational exposure to valproate

**DOI:** 10.3389/fnana.2023.1235047

**Published:** 2023-08-03

**Authors:** Cintia Klaudia Finszter, Róbert Kemecsei, Gergely Zachar, Sophie Holtkamp, Diego Echevarría, István Adorján, Ágota Ádám, András Csillag

**Affiliations:** ^1^Department of Anatomy, Histology and Embryology, Faculty of Medicine, Semmelweis University, Budapest, Hungary; ^2^Institute of Neuroscience (UMH-CSIC), University of Miguel Hernández, Alicante, Spain

**Keywords:** valproate, apoptosis, ASD, calcium binding proteins (CBPs), immunoblotting, synapses

## Abstract

Gestational exposure of mice to valproic acid (VPA) is one currently used experimental model for the investigation of typical failure symptoms associated with autism spectrum disorder (ASD). In the present study we hypothesized that the reduction of dopaminergic source neurons of the VTA, followed by perturbed growth of the mesotelencephalic dopamine pathway (MT), should also modify pattern formation in the dopaminoceptive target regions (particularly its mesoaccumbens/mesolimbic portion). Here, we investigated VPA-evoked cellular morphological (apoptosis-frequency detected by Caspase-3, abundance of Ca-binding proteins, CaBP), as well as synaptic proteomic (western blotting) changes, in selected dopaminoceptive subpallial, as compared to pallial, regions of mice, born to mothers treated with 500 mg/kg VPA on day 13.5 of pregnancy. We observed a surge of apoptosis on VPA treatment in nearly all investigated subpallial and pallial regions; with a non-significant trend of similar increase the nucleus accumbens (NAc) at P7, the age at which the MT pathway reduction has been reported (also supplemented by current findings). Of the CaBPs, calretinin (CR) expression was decreased in pallial regions, most prominently in retrosplenial cortex, but not in the subpallium of P7 mice. Calbindin-D 28K (CB) was selectively reduced in the caudate-putamen (CPu) of VPA exposed animals at P7 but no longer at P60, pointing to a potency of repairment. The VPA-associated overall increase in apoptosis at P7 did not correlate with the abundance and distribution of CaBPs, except in CPu, in which the marked drop of CB was negatively correlated with increased apoptosis. Abundance of parvalbumin (PV) at P60 showed no significant response to VPA treatment in any of the observed regions we did not find colocalization of apoptotic (Casp3+) cells with CaBP-immunoreactive neurons. The proteomic findings suggest reduction of tyrosine hydroxylase in the crude synaptosome fraction of NAc, but not in the CPu, without simultaneous decrease of the synaptic protein, synaptophysin, indicating selective impairment of dopaminergic synapses. The morpho-functional changes found in forebrain regions of VPA-exposed mice may signify dendritic and synaptic reorganization in dopaminergic target regions, with potential translational value to similar impairments in the pathogenesis of human ASD.

## Introduction

Autism Spectrum Disorder (ASD) is one of the most common neurodevelopmental disorders associated with altered social behavior. A cover term for a continuum of failure symptoms from high functioning to seriously defective cases, ASD has been extensively investigated in the recent years both in preclinical and clinical studies. With all its phenomenological variations, impairment of social adaptation and behavior are characteristic features of all forms of ASD, however, its etiology has remained largely elusive. Two main groups of pathogenetic/risk factors (environmental/epigenetic, and genetic) are usually associated with the disease. While only a limited contingent of human autism cases could be traced backed to known genetic components (Scherer and Dawson, 2011; Abrahams et al., 2013), an ever-increasing list of candidate genes, susceptibility factors, and signaling pathways, of diagnostic or predictive value in ASD (Satterstrom et al., 2020), signifies major breakthroughs in the field anytime soon. Still, the bulk of ASD cases are supposed to be brought about by environmental, epigenetic factors exerting their effect at critical time points of embryonic development.

Gestational exposure of laboratory rodents to valproic acid (VPA) is one currently used experimental model for the investigation of typical failure symptoms associated with autism spectrum disorder (ASD). Administration of VPA (a.k.a. 2-propylpentanoic acid), a commonly used anticonvulsant, antiepileptic, and mood stabilizer (Cipriani et al., 2013), to pregnant females elicits characteristic deficits of social behavior in the offspring *post-partum* (Rodier et al., 1996; Schneider and Przewlocki, 2005; Wagner et al., 2006; Kim et al., 2011; Moldrich et al., 2013; Roullet et al., 2013, for comprehensive reviews see Roullet et al., 2013; Nicolini and Fahnestock, 2018). VPA-treated animals also exhibit anxiety, depression-like behavior, and abnormal nociception thresholds (Wu et al., 2017; Mahmood et al., 2018). The validity of embryonic exposure to VPA as a model is not restricted to mammals, as evidenced by recent studies in fish (Baronio et al., 2017; Chen et al., 2018) or birds (Nishigori et al., 2013; Sgadò et al., 2018; Lorenzi et al., 2019; Zachar et al., 2019). The VPA model is often called idiopathic. However, it is relevant to the potential contribution of epigenetic/environmental factors in the pathogenesis of ASD (Ergaz et al., 2016).

Teratogenic effects, including an elevated risk of ASD following administration of VPA (Christianson et al., 1994; Chomiak et al., 2013; Christensen et al., 2013) have long been suggested, largely precluding the use of the agent in pregnant female patients (Tureci et al., 2011). VPA-elicited teratogenic alterations have been described both in chicken embryos (Barnes et al., 1996; Whitsel et al., 2002) and in human clinical studies (Jentink et al., 2010). Most likely, the principal mechanism underlying the autism-like effects is that VPA is an inhibitor of histone deacetylase, modifying transcription (Göttlicher et al., 2001; Kataoka et al., 2013; Moldrich et al., 2013) in a critical time window of embryonic development. When compared to the plethora of behavioral studies, the reports on the anatomical correlates of gestational VPA treatment are rather scarce.

According to our hypothesis, any changes occurring in the offspring *in utero* after VPA administration to the mother, must influence critically timed events of CNS development, and they must also affect those brain regions participating in, or linked to, the social brain network (SBN, Goodson, 2005; O’Connell and Hofmann, 2011, 2012). In previous studies, E12-15 proved to be the appropriate period of VPA administration for eliciting ASD-like behaviors in rats or mice (Kataoka et al., 2013) without major teratogenic consequences. Notably, this time window coincides with the appearance of dopaminergic cells in the brainstem, as revealed by labeling of tyrosine hydroxylase (TH), the rate limiting enzyme of dopamine (DA) synthesis, and the early growth of the dopaminergic axons entering the mesotelencephalic tract (MT). The ventrobasal forebrain, including the nucleus accumbens (NAc), receives dopaminergic input mainly from the ventral tegmental area (VTA), whereas the dorsolateral striatum or caudate-putamen (CPu) receives input mainly from the substantia nigra, pars compacta (SNc) (*cf.* Hasue and Shammah-Lagnado, 2002; Rodríguez-López et al., 2017). Due to its composition and to the extensive connections with limbic forebrain centers (ventral pallidum, extended amygdala, septum, bed nucleus of stria terminalis, olfactory tubercle, prefrontal cortex, and certain hypothalamic nuclei) the ventrobasal forebrain occupies a central position in decision-making based on learned associations, reward, and social attraction (Humphries and Prescott, 2010). Many of these functions are potential targets for impairment due to ASD. The mesolimbic dopaminergic reward system is amply interconnected and overlapping with the SBN (Goodson, 2005) forming the phylogenetically conservative social decision-making network (O’Connell and Hofmann, 2012; Csillag et al., 2022). The latter pathway is also present in birds. Connectivity and chemoarchitecture of these regions, homologous with mammalian progenitor zones, basal ganglia, and extended amygdala (Bupesh et al., 2011; Kuenzel et al., 2011; Vicario et al., 2014, 2015, 2017; Martínez-Cerdeño et al., 2018), have been extensively investigated also by our research group (Bálint et al., 2004; Montagnese et al., 2004, 2008; Hanics et al., 2012, 2017). Lesion to the NAc was found to impair socially motivated vocalization of domestic chicks (Zachar et al., 2017).

A great deal of studies has tackled the pre- and postnatal development of dopaminergic neurons and pathways (see below), the developmental dynamics of the ventrotegmental–accumbens (mesolimbic) pathway, including the chemorepellent or chemoattractant factors specifically involved in the segregation of distinct dopaminergic pathways/connections (see Bissonette and Roesch, 2016).

The suggestion that ASD might be associated with functional impairment (Hamilton et al., 2013), or defective development (Bissonette and Roesch, 2016; Alsanie et al., 2017) of the dopaminergic (DAergic) system, has already been put forward some years ago. Striatal circuits, strongly dependent on DAergic input, have been envisaged as key elements in the development of various forms of ASD (for review see Fuccillo, 2016). Previous reports have raised the possibility that the observed social deficits in VPA-exposed rats might be ascribed to a developmentally determined dysfunction of the dopaminergic system (Schiavi et al., 2019). A high rate of comorbidity of ASD with bipolar disorder, anxiety, and depression (Kirsch et al., 2020) further supports the notion that, at least certain types of autism, are accompanied by dysregulation of the DAergic system. The DAergic system has been implicated in genetic disturbances underlying certain forms of human ASD, including *de novo* missense mutation of the dopamine transporter gene (Hamilton et al., 2013), or alteration of the dopamine-3-receptor gene (DRD3) (Staal et al., 2012), for an overview see Nguyen et al. (2014).

DAergic dysfunction was also verified in animal models of autism (Chao et al., 2020; Kuo and Liu, 2022). Other studies pointed at the importance of the brainstem and related pathways in the pathogenesis of ASD (see Seif et al., 2021). As for the VPA model, recent studies have provided evidence for a key role of DA and DA receptors in the mediation of VPA-related changes of behavior in rats (László et al., 2022), in methamphetamine-evoked hyperlocomotion, mediated by elevation of DA, in the prefrontal cortex of mice (Hara et al., 2015), or in the distribution of mesencephalic DAergic neurons of domestic chicks (Adiletta et al., 2022). Our groups were among the first to demonstrate distinct and coherent neuroanatomical and neurochemical changes in the dopaminergic system of the mouse (Ádám et al., 2020). In juvenile (PD 7) pups of mothers prenatally (ED 13.5) treated with VPA, we reported defasciculation and fiber reduction of the mesotelencephalic tract (Ádám et al., 2020), in apparent agreement with an fMRI study on modified structural and functional connectivity of the mesolimbic reward pathway (linking the ventral tegmental area to the nucleus accumbens and other ventrobasal telencephalic centers) in human subjects (Supekar et al., 2018). In our above-mentioned study, abundance of DAergic neurons was selectively reduced in the ventral tegmental area (VTA) but increased in substantia nigra (SN) (Ádám et al., 2020). In addition, tissue DA concentration was diminished in the NAc, while it remained unchanged in the CPu (Ádám et al., 2020). We then hypothesized that reduced DA level and fiber input at a critical stage of brain development might lead to suboptimal neuronal and synaptic patterning of the DAergic target regions.

The assumption that DAergic innervation might guide the organization of striatal development was formulated about 30 years ago (Voorn et al., 1988). Since then, the ontogeny of relevant dopaminergic centers and pathways has been described in detail, first in rats. On E17 a prominent bundling of DA neurites is taking place (Voorn et al., 1988). In the target zones of striatum, the DA fibers appear first as “patches” (dopaminergic islands) around the time of birth, to get dispersed by the end of week 3. Lack of persistence and failure of continued growth of such islands were found to be critical alterations in the weaver mutant (Roffler-Tarlov and Graybiel, 1987). Polonged growth of DA immunoreactive axons, forming symmetrical axospinous synapses in striatal and accumbal targets (Antonopoulos et al., 2002) indicates that the dopaminergic system may contribute to pattern formation of the entire striatum and NAc. In general, the striatum takes a considerably long time for an adult-like pattern of neuropeptide expression to be attained, and this process requires a synchronous and compartment-selective sequence of afferent innervation (Martin and Cork, 2014). Conversely, altered wiring (derangement of nigrostriatal–to a lesser degree, of ventral tegmentostriatal–axons) was found to precede neurodegeneration in PET studies of human PD (Caminiti et al., 2017).

DA is known to influence the development and maturation of target neurons, as evidenced by co-culture studies (Snyder-Keller et al., 2008). By extrapolation, the ventrobasal forebrain, constituting part of the social brain network, likely undergoes extensive pattern formation regulated by DAergic input. Insufficiency or just retardation of this process (i.e., “missing” the critical time point of embryonic development), may lead to weaker or inadequate response to social signals *post-partum*. Depending on the effectiveness of later compensatory mechanisms, the defect may persist into adulthood, manifested as different forms of ASD.

The point of departure for the present study was the assumption that the reduction of DAergic source neurons of the VTA, perturbed growth and axon guidance of the DAergic pathway (leading to defasciculation), and the selective drop of DA in the NAc, observed in prenatally VPA-exposed animals at P7 (Ádám et al., 2020 and present study) should also interfere with ongoing pattern formation in the dopaminoceptive target regions of the mesotelencephalic pathway (particularly its mesoaccumbens/mesolimbic portion). Accordingly, the main goal of the present study is the investigation of VPA-evoked changes at the level of cells (apoptosis-frequency, abundance and distribution of calcium binding proteins), as well as of synapses (general vs. dopaminergic) in selected dopaminoceptive subpallial regions, as compared to pallial regions.

## Materials and methods

### Experimental animals

For VPA treatment, 6-week-old female C57BL mice with an established pregnancy of 7 days (obtained from Janvier Labs, France) were used. The animals were kept in pairs, with *ad libitum* access to standard laboratory chow and water, under a 12/12 h light/dark cycle. On day 13.5 of pregnancy, one half of the mothers were injected with 500 mg/body weight VPA (diluted at 100 mg/ml with physiological salt) into the occipital skin fold. Control animals were injected with vehicle, under identical conditions. After birth, pups remained with the dams until day 7 (at which time the first experimental groups for immunoblotting or immunohistochemistry were removed). Thereafter, the rest of the pups were separated from the dams (at the age of 4 weeks) and kept together with their littermates until 60 days of age (for long-term immunohistochemical study).

### Immunohistochemistry

Valproic acid-treated and control mice of either sex (16 animals of 7 days, and 6 animals of 60 days of age), were terminally anaesthetized with an intraperitoneal injection of ketamine (50 mg/ml) and xylazine (20 mg/ml) (mixture of 2:1, 0.1 or 0.3 ml total volume, at 7-day-old or at 60-day-old mice, respectively). The animals were transcardially perfused with a solution of 4% paraformaldehyde in PBS, at 4°C. After removal, the brains were post-fixed in the same solution for at least 24 h.

Immunocytochemistry in paraffin-embedded sections. The brains were paraffin-embedded and 10 μm sections were cut in the sagittal or coronal plane, serially mounted on glass slides, and immunostained as performed previously (Moreno-Bravo et al., 2014). The images were collected using a LEICA MZ16FA stereomicroscope equipped with a Leica DFC550 digital camera. For tyrosine hydroxylase (TH) immunostaining, the anti-TH antibody (rabbit polyclonal IgG, Merck Millipore, catalog# AB152) (at 1:500) was used. For neurofilament (NF) immunolabeling, we used the antibody ABCAM AB9034 (1:800).

Immunocytochemistry in free-floating frozen sections. The brains were immersed in 30% sucrose (until they sank to the bottom of the vial) and cut into 30 μm coronal sections with a Leica SM2000R freezing microtome. The sections were stored in 0.01% of azide-containing PBS at 4°C until further processing. Altogether five series of sections were prepared from each brain. From P7 animals, three section series were doubly immunostained for calretinin (CR) and caspase-3 (Casp3), for calbindin (CB) and Casp3, as well as for DARPP-32 and Casp3, respectively. From P60 animals, two section series were singly stained for parvalbumin (PV) or CB. In each case, the technical procedure was similar, always ensuring that the sections from control and VPA-treated animals were handled simultaneously, using the same batch of reagents under the same conditions. The sections were washed for 3 × 10 min, in PBS, treated with H_2_O_2_ (0.3% in PBS, 20 min), washed again for 3 × 10 min, in PBS, and blocked for 30 min in normal goat serum (NGS), 5% in PBS. For those sections processed for DARPP-32, normal horse serum (NHS) was used instead of NGS throughout. Then the sections were incubated with the respective combination of primary antisera (diluted in 0.3% Triton-X-100, PBS), overnight. The primary antibodies were as follows: a-calretinin 1:1,000 chicken (LOT 22024) Thermo Fisher Scientific, a-calbindin 1:1,000 chicken (LOT 22654) Thermo Fisher Scientific, a-caspase-3 1:1,000 rabbit (LOT CN89330), Bioworld Technology, a-parvalbumin 1:1,000 chicken (LOT 22579) Thermo Fisher Scientific, a-DARPP-32 1:1,000 goat (LOT XHG0320061), RD Systems. Following washing for 3 × 10 min, in PBS, the sections were incubated with the respective ‘ary antibodies, (Goat a-chicken, Alexa Fluor 594 (LOT WA316328) 1:1,000, Invitrogen; Donkey a-rabbit, Alexa Fluor 488 (LOT VC296619) 1:1,000, Invitrogen), for 1 h, followed by another washing for 3 × 10 min, in PBS. The sections were mounted on glass slides and coverslipped using a glycerol/PBS mixture (1:1).

### Microscopy and cell counting

For cell counting studies, the specimens were viewed and photographed under a Nikon Eclipse E800 fluorescence microscope at 10x, 20x, or 40x magnifications. In the reference frames, marked out according to the coordinates of the atlas by Paxinos and Franklin (2001), all labeled cells (in case of double labeling, separately for each fluorochrome) were counted manually, in representative coronal sections of each brain, using the Aperio ImageScope software. The values were expressed as number of cells per mm^2^. We performed manual counting throughout, including manual thresholding. The latter meant that the diameter of each labeled cell to be counted was measured using the feature of the image viewer program (Aperio ImageScope) of the fluorescent microscope. Only those cell bodies larger than 5 μm in longest diameter were counted. Counting and further analysis was done on photographic images off-line, the images were coded by one observer and counted blind by another colleague, who was not aware of the code until the end of the analysis.

**FIGURE 1:**
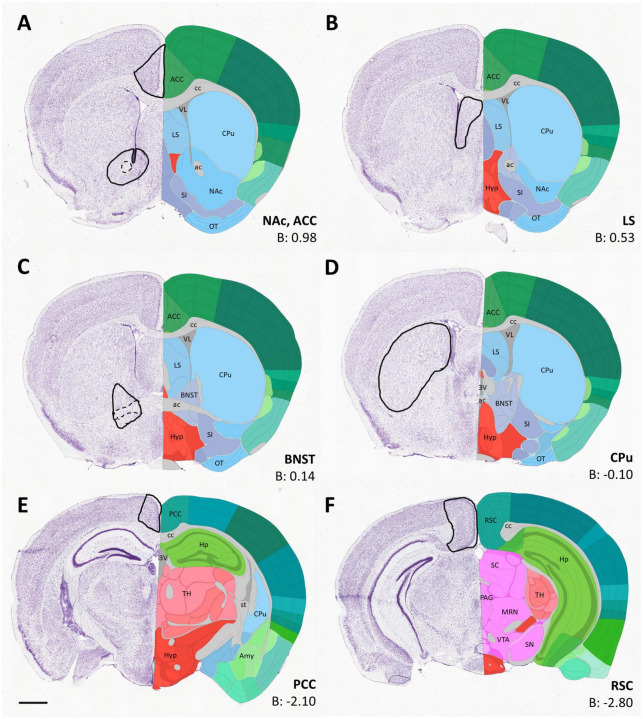
Diagrams **(A–F)** depicting the location of sampling frames for cell counting for each region of interest (indicated in bold next to the respective diagram), applied consistently for every brain measured. The coronal images were adapted from the Allen Brain Atlas (https://atlas.brain-map.org/). The outlines of the measuring frames are projected onto the corresponding atlas image (left side), with some key landmarks (right side) and AP coordinates. The areas delineated by dashed lines were excluded from sampling. 3V, third ventricle; ac, anterior commissure; ACC, anterior cingulate cortex; Amy, amygdala; B, Bregma; BNST, bed nucleus of stria terminalis; cc, corpus callosum; CPu, caudate-putamen; EP, entopeduncular nucleus; Hp, hippocampus; Hyp, hypothalamus; LS, lateral septum; MRN, midbrain reticular nucleus; NAc, nucleus accumbens; OT, olfactory tubercle; PAG, periaqueductal gray; PCC, posterior cingulate cortex; RSC, retrosplenial cortex; SC, superior colliculus; SI, substantia innominata; SN, substantia nigra; st, stria terminalis; TH, thalamus; VL, lateral ventricle; VTA, ventral tegmental area. Scale bar: 1 mm.

Concerning the sampling procedure, single measuring frames were superimposed to representative coronal sections adapted from The Allen Mouse Brain Atlas (© 2021 Allen Institute for Brain Science. Mouse Brain Connectivity),^1^ as defined consistently for each region of interest (ROI), at identical A/P levels in every brain. Both hemispheres were used for counting, but the values were not combined, since, in some cases, only one hemisphere remained intact. Rather than using regular quadrangles, the measuring frames for each ROI were marked out by hand, to better fit to the boundaries of nuclei, to avoid noise from artifacts and to exclude non-relevant areas, such as brain pathways (e.g., the anterior commissure, in the case of NAc). The relevant anatomical landmarks and stereotaxic coordinates defining the sectional level, as well as the boundaries for each ROI, are summarized by diagrammatic drawings depicting the location of the measuring frames for every brain region analyzed (Figure 1).

Of the pallial regions, the anterior cingulate cortex (ACC), posterior cingulate cortex (PCC) and retrosplenial cortex (RSC) were analyzed. Regarding subpallial regions, we quantified the number of cells in lateral septum (LS), nucleus accumbens (NAc), bed nucleus of stria terminalis (BNST), and caudate-putamen (CPu).

Statistical analysis of results was based on individual hemispheric data (with the animals as random factor), using the R Studio program. To determine the effects of treatment (VPA vs. control) and age (7 vs. 60 days) as fixed effects, we applied a generalized linear mixed model (GLMM) based on negative binomial distribution with a Log link function^2^ in every brain region. The linear mixed model included the data of the two hemispheres separately to control for within-subject variance but, since the ID of the individuals was included as a random effect, the two hemispheres’ data were NOT treated as statistically independent. Accordingly, the datapoints on the graphs represent the cell density values of the given brain region from unilateral hemispheres, rather than the mean value of those regions from bilateral hemispheres.

Although the design of the experiment was not intended to specify sex differences, the sex of the individuals was also included in the model in order to control for it. In most cases, the gender of the animals neither had any significant effect, nor was it included in any significant interaction (expect for one specific case mentioned below).

For high-resolution microscopic analysis of colocalization, one group of the mounted specimens were viewed and photographed under a LSM 780 confocal laser microscope (Zeiss Jena, Germany), the images were stitched using the ZEN 2010 program. The degree of cellular overlapping was assessed on Z-stacks. For analysis of another contingent of specimens an Olympus Fluoview FV3000 confocal laser scanning microscope was used, applying the Olympus FV31S-SW image processing software.

For quantification of cellular overlapping, confocal image stacks were analyzed using an Imaris software package (Bitplane AG, Switzerland, version 9.9.1.) operating on a HP Z4 control system. The “Coloc” feature of Imaris implements the Costes and Lockett method (Costes et al., 2004), which automates selection of colocalized voxels, thereby eliminating operator-related bias, while providing analysis and visualization of colocalization in multi-channel datasets. Colocalization of Casp3 with CR was determined on a per-pixel basis.

### Immunoblotting (western blot)

**FIGURE 2:**
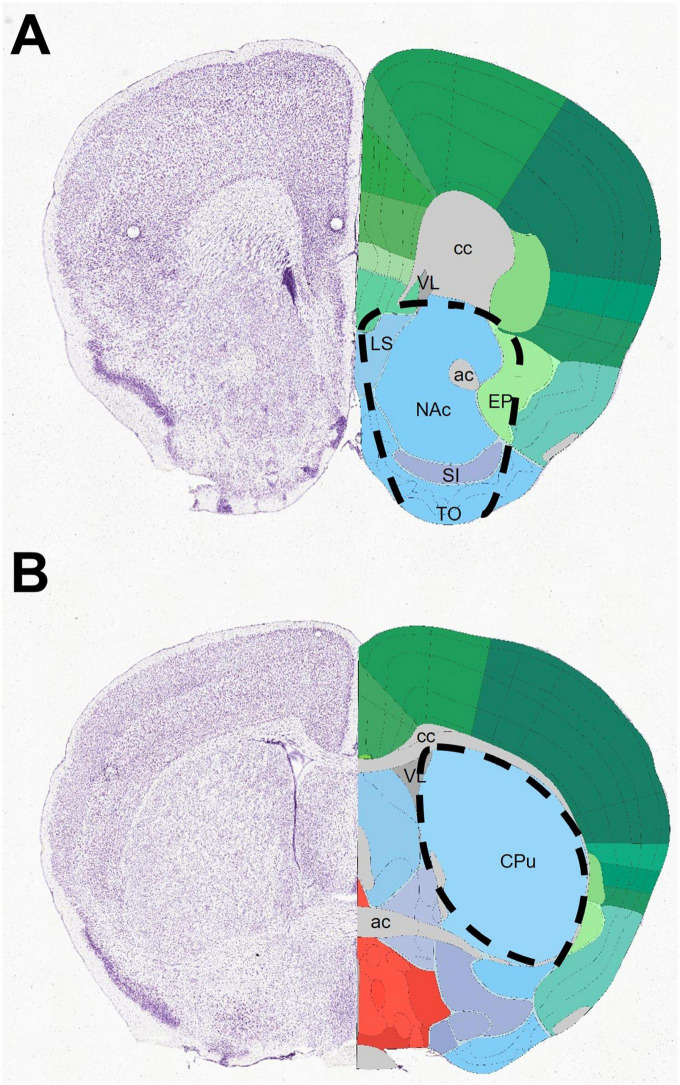
Diagrams depicting the dissection of specimens, centered on the nucleus accumbens **(A)** or caudate-putamen **(B)**, collected for western blotting. The rostral facet of tissue slabs corresponds to coronal images 39 **(A)** and 53 **(B)** of the mouse brain from the Allen Brain Atlas (https://atlas.brain-map.org/). The approximate outlines of dissected regions are projected onto the corresponding atlas image. ac, anterior commissure; cc, corpus callosum (anterior forceps); CPu, caudate-putamen; EP, entopeduncular nucleus; LS, lateral septum; NAc, nucleus accumbens; SI, substantia innominata; TO, olfactory tubercle; VL, lateral ventricle.

Twenty-two 7-day-old mouse pups of either sex was used. Immediately after decapitation of the animals, the brains were removed and dissected into coronal slabs with the help of a steel brain matrix for reproducible blocking. Further dissection of the slabs was carried out on top of a black anodized aluminum box containing ice, under a stereomicroscope. Tissue samples of 10–20 mg wet weight from the NAc and CPu were dissected bilaterally. The diagrams in Figure 2 show the rostral facet of the cut slab with the approximate borders of dissection projected onto the respective images of the Allen Brain Atlas. For tissue localization, we consulted The Allen Mouse Brain Atlas (© 2021 Allen Institute for Brain Science. Mouse Brain Connectivity) (see text footnote 1). The removed tissue blocks of NAc contained major parts of the NAc (practically the whole core and most of the shell), together with fragments of the adjacent septum, bed nucleus of stria terminalis, part of the entopeduncular nucleus, substantia innominata, olfactory tubercle and the anterior commissure. In addition to the striatal mass, the tissue blocks of CPu also contained parts of the pallidum externum (GPe) together with fibers of the internal capsule.

The dissected brain specimens (the combined bilateral samples counted as one) were immediately placed into pre-weighed Eppendorf tubes and stored in dry ice until further processing. The samples were homogenized in ice-cold lysis buffer (150 mM NaCl; 1% Non-idet P-40; 0.5% sodium deoxycholate; 0.1% sodium dodecyl sulfate, SDS; 50 mM Tris, pH 8.0). To separate the cellular debris from total protein content, centrifugation at 4°C, 1,200 *g* for 10 min followed. The supernatant was centrifuged again at 15,000 *g* for 20 min at 4°C. The sediment, containing the crude synaptosome fraction, was resuspended according to the wet weight, Then, all samples were diluted to an even protein concentration (1.25 μg/μl), as determined by the BCA method (Olson and Markwell, 2007) with the help of a Bio-Rad iMark Microplate Reader. The specimens were dissolved in Laemmli buffer (Sigma-Aldrich) and heated at 96°C for 5 min. Samples were loaded first onto 8% acrylamide gel (stacking gel) at 30 V, 20 min, followed by 13% resolving gel (150 V, 1–1.5 h), and processed with a Bio-Rad Mini-Protean III vertical electrophoresis and blotting system. Then the separated samples were electrophoretically transferred onto nitrocellulose membranes (Bio-Rad, 0.45 μm pore size, 90 V, 2 h, under external cooling). For immunoblotting, the membranes were incubated with 5% non-fat dry milk in TBS-T buffer (0.05 M Tris-buffered saline pH 7.4 and 0.1% Triton X) for 1 h at room temperature. Then, these were incubated with the following cocktail of primary antibodies: a-synaptophysin, mouse, DAKO 1:1,000; a-tyrosine hydroxylase, rabbit, EMD Millipore, 1:5,000, a-ß-actin, mouse, Cell Signaling Technology, 1:10 000) overnight at 4°C. After washing, the samples were treated with the following cocktail of secondary antibodies: Donkey a-mouse Alexa Fluor 594, Molecular Probes, 1:1,000; Donkey anti-rabbit Alexa Fluor 488 Invitrogen, 1:1,000. The blots were visualized and quantified with an enhanced luminescence detection system (Bio-Rad ChemiDoc MP) and standardized by the luminosity of the housekeeping protein ß-actin, as loading control, using ImageLab software.

## Results

**FIGURE 3:**
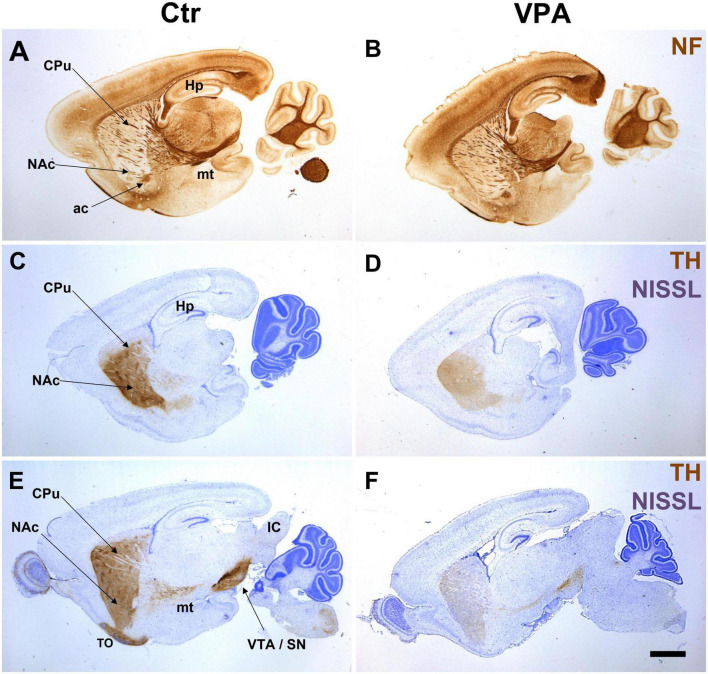
Representative series of paraffin-embedded sagittal sections from control (Ctr) and VPA-exposed (VPA) P7 mice immunolabeled for neurofilament protein [NF, images **(A,B)**], or tyrosine hydroxylase [TH, Nissl counterstaining, images **(C–F)**]. No overall difference between Ctr and VPA is evident in the morphological appearance of the mesotelencephalic tract (mt) in the NF specimens. Conversely, TH labeling appears sparser and more dispersed in the mt tract and its target regions in the specimens from VPA-exposed mice as compared to those from control mice. Note the conspicuous lack of TH label in the olfactory tubercle (**E** vs. **F**). ac, anterior commissure; CPu, caudate-putamen; Hp, hippocampus; NAc, nucleus accumbens; TO, olfactory tubercle; VTA/SN, ventral tegmental area/substantia nigra. Scale bar: 1 mm.

In agreement with the previously reported findings (Ádám et al., 2020), a marked reduction of staining intensity, and a visible widening and dispersion of TH + structures along the path of MT were observed in VPA-exposed pups (Figures 3C, D). One prominent alteration, not emphasized previously, was the virtual disappearance of TH labeling at the olfactory tubercle (Figures 3E, F). Notably, the morphological appearance (size, course, fiber packing) of the MT, in general, remained unchanged, as evidenced by NF staining (Figures 3A, B), extending the validity of our previous observation to the present experimental group of mice.

**FIGURE 4:**
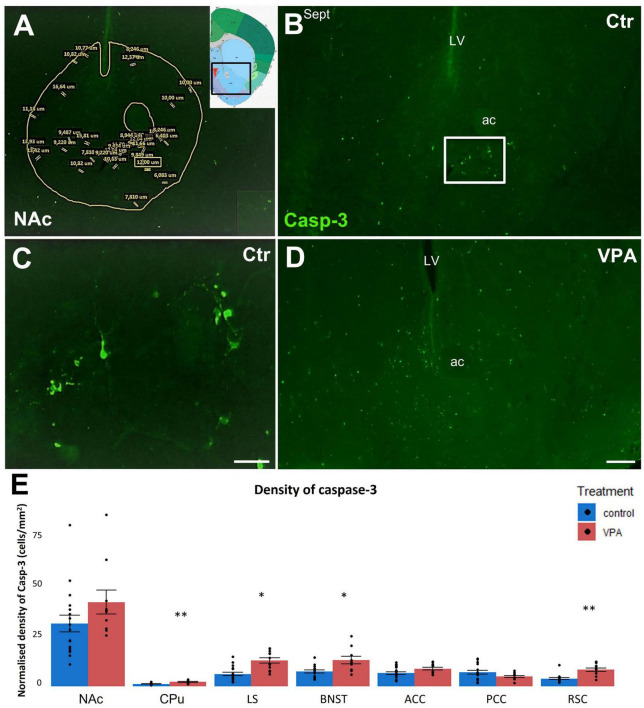
**(A–D)**: Representative fluorescence microscope images demonstrating the distribution of apoptotic (Casp3 +) cells of P7 mice born to VPA-treated (VPA) to vehicle-treated (Ctr) mothers. A typical example for the segmentation of the region of interest (ROI) on each tissue section (here the NAc) is demonstrated on image **(A)**, the inset shows the position of this region on the reference atlas figure. Within the ROI (areas of tracts excluded), all Casp3 + elements which met the preset shape and size criteria (the size values are displayed here for demonstration purpose only) were counted as apoptotic cells. Image **(B)** shows the accumulation of apoptotic cells in the NAc of control mouse (Ctr), the framed area appears under higher magnification in Image **(C)**, for better visualization of the neuronal architecture of Casp3 + structures. Compared to control, apoptotic cells appear more abundant in the same region of the VPA mouse [Image **(D)**]. Notably, due to high variability, this prominent trend failed to attain significance level in the NAc, whereas, in most other regions, the VPA-associated surge of apoptosis was significant. Abbreviations on images: ac, anterior commissure; Sept, septum; LV, lateral ventricle. Scale bars: 200 μm **(B,D)**, 50 μm **(C)**. The attached graph **(E)** shows the areal density of Casp3 + cells in several subpallial and pallial regions, comparing VPA-exposed (*n* = 10–12) and control (*n* = 17–20) hemispheres (depending on the intactness of given brain regions), representing 6 and 10 animals, respectively. The values are expressed as M ± S.E.M. *p < 0.05, ^**^p < 0.01. NAc, nucleus accumbens; CPu, caudate-putamen; LS, lateral septum; BNST, bed nucleus of stria terminalis; ACC, anterior cingulate cortex; PCC, posterior cingulate cortex; RSC, retrosplenial cortex.

To specify the nature of potential sequelae of impaired DAergic input, first, we analyzed neuronal degeneration (apoptosis) in the NAc (main target of the mesolimbic MT), parallel with other subpallial and pallial regions, by quantitatively measuring the areal density of the perikarya expressing Casp3, a common apoptosis marker enzyme (Jänicke et al., 1998), comparing VPA-exposed (VPA) and vehicle-exposed (control, CTR) mice (Figures 4A-D).

In P7 mice, the overall level of apoptosis was expected to be high, since this period corresponds to the second (postnatal) apoptotic surge in the CNS (Southwell et al., 2012; Dekkers et al., 2013; Nikolić et al., 2013). In our study, the regional density of apoptotic cells (as detected by immunoreactivity to Casp3) was increased in the VPA-exposed group in several regions detected (Figure 4E). In subpallial regions, the lateral septum (LS), bed nucleus of stria terminalis (BNST), and caudate-putamen (CPu) showed marked elevation of activated Casp3, and the trend was similar, though not significant, in the NAc. Of the pallial regions, increase of the density of Casp3 + cells was highly significant in the retrosplenial cortex (RSC), but not significant in the anterior cingulate cortex (ACC), and the posterior cingulate cortex (PCC).

**FIGURE 5:**
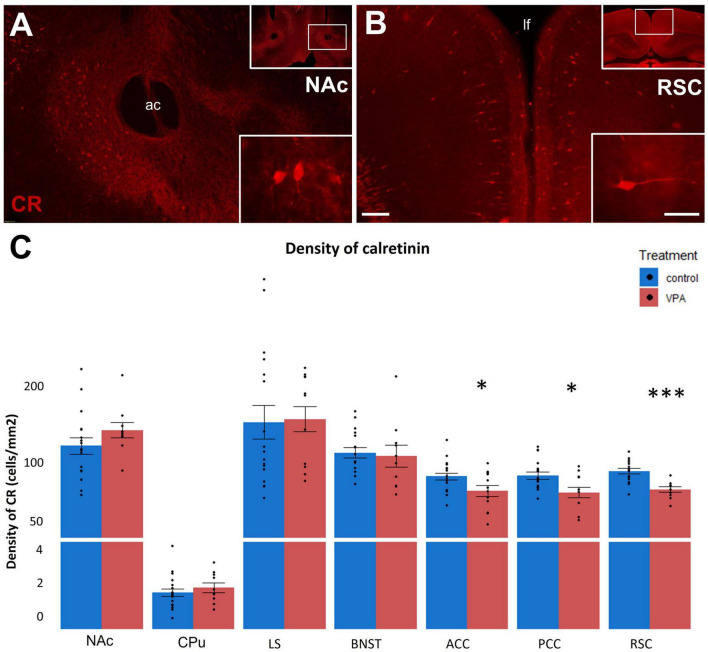
**(A,B)**: Fluorescence microscope images exemplifying the distribution of cells immunolabeled for calretinin (CR) in a subpallial (NAc) and a pallial (RSC) region of control P7 mice. The framed areas on the upper right insets are displayed under intermediate magnification (Scale bar: 100 μm). Characteristic cell types of the region are shown under high magnification (Scale bar: 20 μm) on the lower right insets. Abbreviation on images: ac, anterior commissure; lf, longitudinal fissure. The enclosed graph **(C)** shows the areal density of CR-positive perikarya in several subpallial and pallial regions, comparing VPA-exposed (*n* = 10–12) and control (*n* = 17–20) hemispheres (depending on the intactness of given brain regions), representing 6 and 10 animals, respectively. The values are expressed as M ± S.E.M. *p < 0.05, ^***^p < 0.001. Abbreviations as in Figure 4E.

Next, we analyzed the distribution of calcium binding proteins (CaBP) in the above subpallial and pallial regions in VPA-exposed and control P7 mice. Given the observed alterations in apoptosis, it was reasonable to expect changes also in the expression of these, potentially neuroprotective, proteins. The density of calretinin (CR; Figures 5A, B) immunoreactive cells did not show any significant difference in the subpallial regions NAc, LS, BNST and CPu, whereas it was markedly decreased in the cingulate cortex (ACC and PCC) and, most prominently, in the retrosplenial cortex (RSC) (Figure 5C).

**FIGURE 6:**
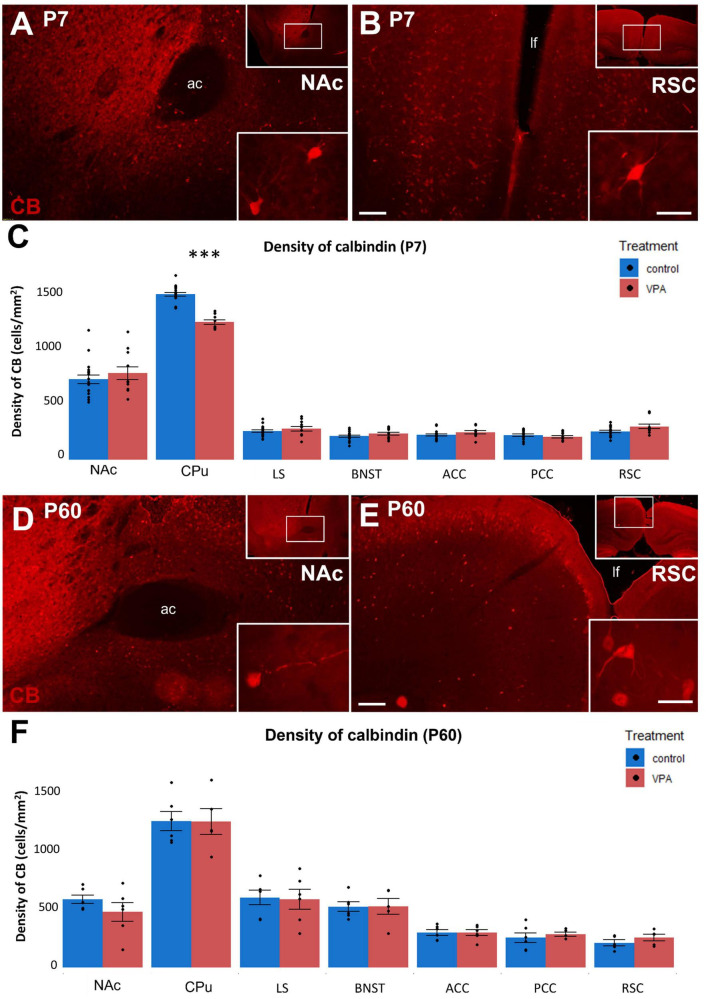
**(A,B,D,E)**: Fluorescence microscope images exemplifying the distribution of cells immunolabeled for calbindin (CB) in a subpallial (NAc) and a pallial (RSC) region of control P7 **(A,B)** and P60 **(D,E)** mice. The framed areas on the upper right insets are displayed under intermediate magnification (Scale bar: 100 μm). Characteristic cell types of the regions are shown under high magnification (Scale bar: 20 μm) on the lower right insets. Abbreviation on images: ac, anterior commissure; lf, longitudinal fissure. The enclosed graphs **(C,F)** show the areal density of CB-positive perikarya in several subpallial and pallial regions, comparing VPA-exposed and control animals. For P7 mice, *n* = 11–12 hemispheres, representing 6 animals (VPA); *n* = 18–20 hemispheres, representing 10 animals (control). For P60 mice, *n* = 6 hemispheres, representing 3 animals (VPA); *n* = 6 hemispheres, representing 3 animals (control). The values are expressed as M ± S.E.M. ^***^p < 0.001. Abbreviations as in Figure 4E.

Conversely, the abundance of calbindin-D 28K (CB; Figure 6) was not changed by VPA exposure in any of the regions, except for the CPu, where it was significantly reduced, when observed at P7 (Figures 6A-C). Remarkably, in P60 animals, even those effects disappeared (Figures 6D-F). When comparing the abundance of CB + neurons in juvenile and adult mice (control values), the highest overall density values (above 1,000/mm^2^ were detected in the CPu (in both age groups), and the largest between-age differences were found in the LS and BNST (2.5-3-fold increase with age), whereas in the CPu and NAc the values tended to drop down at P60 by ca. 15%. Based on further statistical analysis, the age had a significant effect (*p* < 0.05) on the CB cell density in all of the brain regions observed. Furthermore, age and VPA treatment also had a significant interaction in the CPu (*p* = 0.01). Notably, the only significant effect of sex observed in the present dataset of cell density values was an interaction with the age in the case of CB in the LS (*p* = 0.013).

**FIGURE 7:**
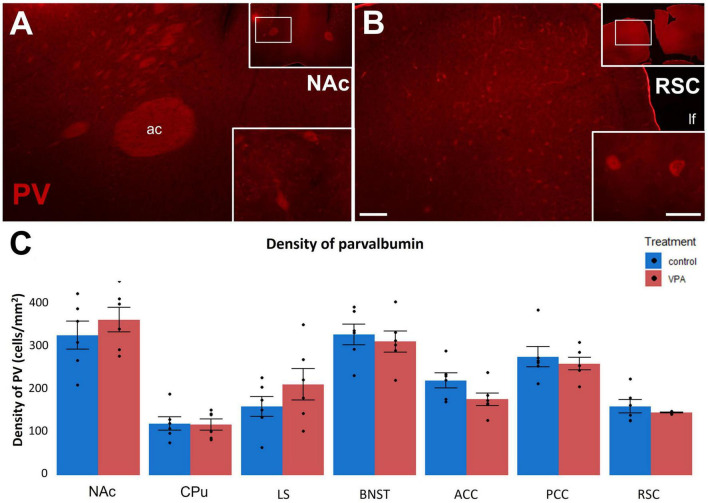
**(A,B)**: Fluorescence microscope images exemplifying the distribution of cells immunolabeled for parvalbumin (PV) in a subpallial (NAc) and a pallial (RSC) region of control P60 mice. The framed areas on the upper right insets are displayed under intermediate magnification (Scale bar: 100 μm). Characteristic cell types of the region are shown under high magnification (Scale bar: 20 μm) on the lower right insets. Abbreviations: ac, anterior commissure; lf, longitudinal fissure. The enclosed graph **(C)** shows the areal density of PV-positive perikarya in several subpallial and pallial regions, comparing VPA-exposed mice (*n* = 6 hemispheres, representing 3 animals) and control (*n* = 6 hemispheres, representing 3 animals). The values are expressed as M ± S.E.M. Abbreviations as in Figure 4E.

Concerning the third CaBP to be studied, parvalbumin (PV) is still absent in P7 mice and appears only later in development (Lauber et al., 2016). At P60 the control density values ranged between 160 and 260/mm^2^ in the cingulate and retrosplenial (Figure 7B) cortices, around 300/mm^2^ in the NAc (Figure 7A) and BNST, 150–200/mm^2^ in LS, and 90–140/mm^2^ in the CPu. None of these regions showed a significant response to VPA treatment (Figure 7C).

**FIGURE 8:**
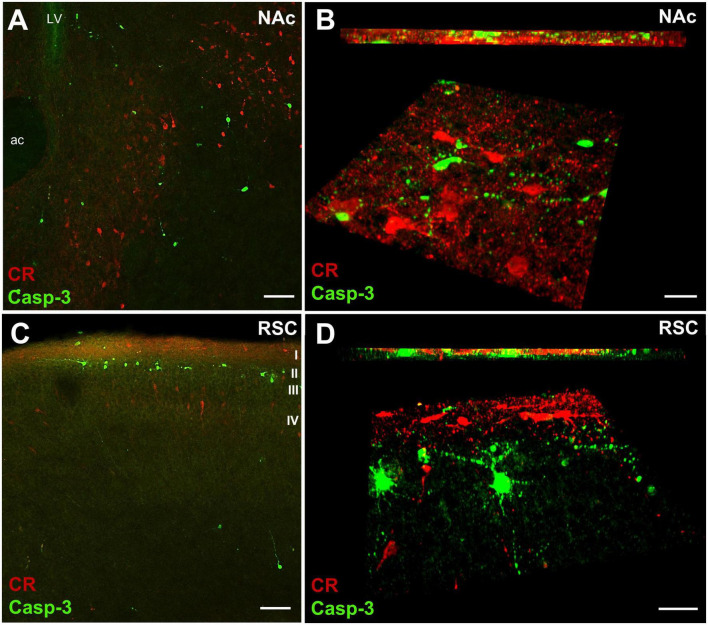
Representative confocal laser scanning images demonstrating non-colocalization of CR and Casp3 immunoreactive neurons in the core of nucleus accumbens (NAc) and in the retrosplenial cortex (RSC) of P7 control mouse. **(A,C)** Overview images, **(B,D)**: high magnification quasi-3D reconstruction (lower panels) and Z stack (upper panel) images rendered with the help of Imaris image analysis software. A high degree of spatial overlap is evident between the two labels in the NAc core **(A)**. Casp3 + and CR + perikarya appear spatially more separated in the RSC, the former being mainly in lamina 2 of cortex, and the latter in lamina 1, or in deeper infragranular layers **(C)**. However, such separation of perikarya does not preclude an overlap of neuronal processes. As apparent from the 3-D and Z stack images **(B,D)**, the two labels are predominantly present in separate, non-overlapping neuronal elements. Scale bars: 50 μm **(A,C)**, 30 μm **(B,D)**. LV, lateral ventricle; Approximate position of cortical layers is marked by Roman numerals I-IV.

**FIGURE 9:**
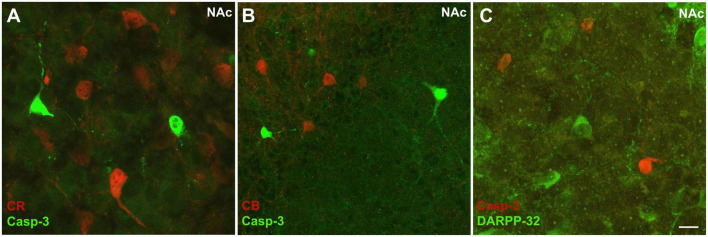
Representative high magnification confocal laser scanning images demonstrating non-colocalization between Casp3 immunolabeled perikarya and those immunoreactive to calretinin **(A)**, calbindin **(B)**, or DARPP-32 **(C)** in the NAc of P7 control mouse. Notably, for combined immunostaining with DARPP-32, for technical reasons, CR was labeled here with the red fluorochrome **(C)**, unlike in the previous examples. Scale bar: 20 μm.

We did not find considerable overlap between Casp3 + and CR + structures in either the NAc (Figures 8A, B) or the RSC (Figures 8C, D), in P7 rats, the quantitative rate of colocalization being 0.02% and 0.09% in NAc and RSC, respectively. Moreover, as detected in the NAc (Figure 9) or CPu (not shown), Casp3 + cells failed to colocalize not only with CR (Figure 9A), but also with CB (Figure 9B) or even with DARPP-32 immunoreactive perikarya (Figure 9C). This means that, so far, we have not been able to pinpoint a particular category of interneuron or principal projection neuron to undergo apoptosis under the given conditions. This problem will require further investigation.

**FIGURE 10:**
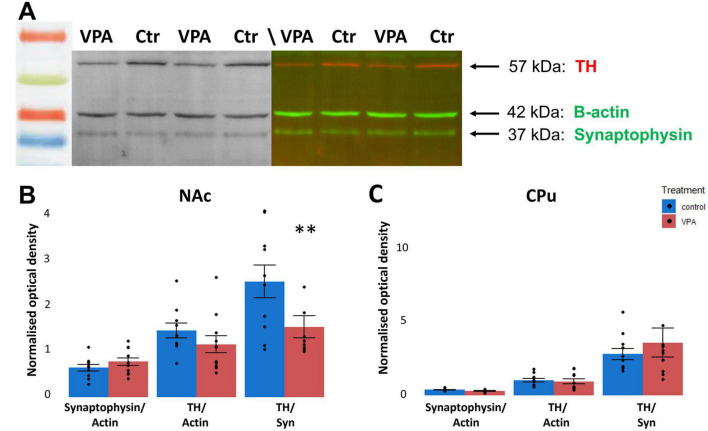
Proteomic analysis (WB) of TH protein relative to synaptophysin in the NAc of VPA-treated animals, compared to controls. The top panel **(A)** shows representative tracks of gels doubly labeled against TH (red) and synaptophysin (green), allowing for direct comparison. The housekeeping protein β-actin (also appearing green) was used for loading control. The diagrams on the bottom panel **(B,C)** show quantitative data of the systematic comparison between control and VPA-exposed specimens from the NAc or the CPu. The data are expressed as M ± S.E.M. *n* = 11 brains (VPA), *n* = 11 brains (control). ^**^p < 0.01.

Further to cellular changes, we searched for alterations of synaptic development by comparing two dopaminoceptive regions of subpallium: CPu and NAc. Proteomic investigation (western blotting) of tissue samples harvested from 7-day-old mouse pups born to VPA-treated or vehicle-treated mothers was focused on simultaneous detection of synaptophysin and TH (Figure 10A). We found that the concentration of synaptophysin (Syn, normalized by the housekeeping protein β-actin) remained stable both in the NAc (Figure 10B) and CPu (Figure 10C), whilst TH (normalized by β-actin) did not change in the CPu but it did show a trend of decrease in NAc (Figures 10B, C). When normalized by Syn, the TH/Syn ratio was markedly reduced in the NAc (Figure 10B), but not in the CPu (Figure 10C), of the VPA-exposed group.

## Discussion

The findings point at a general increase of apoptosis on VPA treatment; although no significant increase was detected in the NAc, the most exposed target of the MT, the direction of the difference does not contradict the general trend. It is worth considering that, in previous studies (Ádám et al., 2014), the greatest density of Casp3 + neurons were observed in the NAc, in P7 control mice. Perhaps, this explains, why we could not detect a significant VPA-associated elevation of apoptosis against an already high baseline (also confirmed in the present study).

Concerning the density and distribution of CaBPs, the corollary from the findings is that at P7, the age at which the MT pathway reduction was observed (Ádám et al., 2020) and enhanced apoptosis on VPA seems to occur in nearly all investigated subpallial and pallial regions (current study), CR is decreased in pallial (cortical) regions but not in the subpallium. Conversely, in a recent report on adult (3-month-old) mice dopamine-depleted by 6-OHDA lesion, the density of medium-sized CR interneurons was decreased in the striatum but not in the NAc (Petryszyn et al., 2021). The latter report lends support to the protective role of CR in the specific case of dopamine depletion (modeling Parkinson’s disease), and so does our current finding on elevated apoptosis concurrent with reduced CR in the RSC. However, it remains difficult to compare surgical or pharmacological treatments of postnatal animals (i.e., taking out DA from an established neuronal circuitry) with a gestational challenge to VPA, leading to developmental weakening or reduction of the DAergic input pathway. In a previous study comparing postmortem human brain samples of normally developing and autistic individuals (Adorjan et al., 2020), the abundance of CR cells was found to be reduced in the caudate nucleus. In our VPA model, a reduction of CR was also observed but only in cortical regions, most prominently in the RSC (which was not analyzed in the cited report). Presumably, the different experimental conditions (among others, age differences) do not allow direct comparison of the studies, even if there may be promising points of convergence.

Calbindin is selectively reduced in the CPu of VPA exposed animals at P7 but no longer at P60. Thus, the VPA-associated overall increase in apoptosis at P7 does not seem to correlate with the abundance and distribution of CaBPs (PV is absent at this age, anyway). The only exception is CPu, in which the marked drop of CB may be negatively correlated with increased apoptosis. This may well reflect a protective effect of CB, apparently getting compromised in the striatum by gestational VPA exposure, at least by the age of P7. It is of interest, however, that the observed drop in CB in CPu disappears by P60, which may reflect an increased resilience of the region. Thus, an early detrimental effect can, visibly, be restored. This is further supported by the apparent stability of PV, not responding to gestational VPA exposure at P60, whereas, at P25 (i.e., nearer to the onset of PV expression) a distinct drop of the number of PV + cells was reported in the striatum (but not in the cortex) of mice born to VPA exposed dams (Lauber et al., 2016). It is worth to be noted that, as mice approach adulthood, their sociability is often decreased (Moy et al., 2004; Ferri et al., 2020; Fereshetyan et al., 2021), or at least its focus and patterns are changed, probably due to sexual maturation. Such a maturation in behavior most likely follows alterations of neural background. The decrease of CB expression in healthy mice between P7 and P60, might be one such alteration. The similar CB cell density in the VPA-treated and control mice at P60 might correspond to lower sociability. Even when there is no apparent change of CB expression in the adult mice, the social development of the young can still be affected by an altered expression, evident only during the first weeks of age, resulting in permanent changes in other behavioral and physiological traits.

The non-overlapping distribution of Casp3 + cells vs. CaBP-immunoreactive neurons, observed in the present study, is in apparent agreement with Lema Tomé et al. (2006), reporting on MK801-induced activated caspase-3 expression most frequently found in mutually exclusive cell populations to those expressing CaBPs.

The proteomic findings suggest selective reduction of TH in NAc, in agreement with our previous histological and neurochemical data (Ádám et al., 2020) without simultaneous decrease of the synaptic protein, synaptophysin. Since TH + perikarya are extremely rare in striatal regions, we assume that most of the TH protein detected there is proportional to the abundance of TH + (largely dopaminergic) axons and synaptic terminals. The value of synaptophysin is proportional to the total synaptic surface, indirectly representing the number of synapses. Previous studies on synaptic changes in ASD models have yielded controversial results. Increased dendritic spine density and enhanced neuronal arborization have been found in the NAc and ventral hippocampus, together with a decreased spine density in the dorsal hippocampus or basolateral amygdala in VPA-exposed rats (Bringas et al., 2013). Reduced spine density together with a decrease in the expression of the mRNAs of synaptic proteins (PSD 95, Shank3) were reported by Yamaguchi et al. (2017) in the hippocampus of VPA-exposed mice. Given the different brain regions and ages (in the cited reports adolescent or adult animals were used), it is difficult to compare these results with our current findings. However, in general, the previous studies seem to corroborate the existence of meaningful synaptic reorganization in limbic forebrain regions of VPA-exposed animals. Enhanced dendritic arborization and spinogenesis (observed in the NAc) likely represent reduced or defective pruning in the affected regions (Bringas et al., 2013). The selective reduction of TH in NAc, but not in CPu (present study) invites a speculation that limbic disruption and hyperactivity in ASD syndrome may be associated or even caused by misdirection of tegmental dopaminergic fibers from ventral (limbic) to dorsal striatum.

At variance with our findings (Ádám et al., 2020, present study), no difference was found in either the abundance of DAergic neurons of SN or VTA, or the expression of TH in CPu or NAc, in mice prenatally exposed to VPA (Maisterrena et al., 2022). The apparent controversy may be due to the different age of animals (P7 in our study, and P21 in the cited study), and to methodological differences in preparing samples for WB. The cited authors used whole lysates for WB, whereas we prepared crude synaptosome fraction from the primary lysate, which, in our opinion, represents synaptic and membrane-bound TH more specifically. Interestingly, Maisterrena et al. (2022) reported an upregulation of DA receptors, indicating an underlying reduction of ligand (DA), which may have had occurred (unnoticed, in that case) prior to the age (P21) at which their samples for immunoblotting were collected. Similarly, upregulation of first D2 (at P35-40), then D1 receptors (at P90-95) was observed in the NAc of rats born to VPA-exposed mothers, (with no data on P7 animals), by Schiavi et al. (2019), together with characteristic behavioral and electrophysiological responses. Both above results can be interpreted as compensatory receptor upregulation following earlier deprivation of ligand (i.e., DA), as indeed it was detected by us in P7 mice.

According to recent findings, elevation of Casp3 may not necessarily be followed by neuronal death. Such non-apoptotic Casp3 is thought to regulate dendritic regression and the pruning of dendritic spines (Ertürk et al., 2014). In a recent study, activation of Casp3 in striatal projection neurons was found to concur with spine loss and defective LTD following experimental degeneration of the nigrostriatal DAergic pathway of mice (Fieblinger et al., 2022). Similar alterations may occur also in the target regions of the mesolimbic DAergic pathway (such as the NAc), albeit, in the present study, we did not find significant elevation (just a trend) of Casp3 in the NAc, of all regions. Nevertheless, the Casp3 activation found in most subpallial, and pallial brain regions of VPA-exposed mice may signify dendritic and synaptic reorganization during a sensitive period of social development after birth, as distinct from cellular degeneration, concurrent with the developmentally determined reduction of DAergic input.

## Data availability statement

The raw data supporting the conclusions of this article will be made available by the authors, without undue reservation.

## Ethics statement

The animal study was reviewed and approved by the Food Chain Safety and Animal Health Directorate of the Government Office for Pest County, Hungary (XIV-I-001-2269-4/2012).

## Author contributions

CF, RK, and SH carried out the experiments and histological processing of specimens. CF, RK, SH, and ÁÁ contributed to the microscopy and quantitative analysis. CF and RK performed the immunoblotting. IA and ÁÁ acted as Ph.D supervisors for the experiments by CF. GZ and DE participated in the design and evaluation of experiments, and in the writing of manuscript. AC conceptualized the study and wrote the first version of manuscript. All authors contributed to the article and approved the submitted version.

## Footnotes

1
https://connectivity.brain-map.org

2
https://www.ncbi.nlm.nih.gov/pmc/articles/PMC6661358/
